# Which deliberate self-poisoning patients are most likely to make high-lethality suicide attempts?

**DOI:** 10.1186/s13033-015-0028-4

**Published:** 2015-09-07

**Authors:** Sang Hoon Oh, Han Joon Kim, Soo Hyun Kim, Young Min Kim, Kyu Nam Park

**Affiliations:** Department of Emergency Medicine, Seoul St. Mary’s Hospital, College of Medicine, The Catholic University of Korea, 222 Banpo-daero, Seocho-gu, Seoul, 137-701 Republic of Korea

**Keywords:** Deliberate self-poisoning, Risk factors, Rescue factors, Suicide attempts

## Abstract

**Background:**

The risk/rescue rating scale (RRRS) assesses the lethality of a suicide attempt, which is defined as the probability of inflicting irreversible damage. We assessed the lethality of suicide attempts using the RRRS and identified the socio-demographic profiles of patients who achieved high lethality in deliberate self-poisoning (DSP).

**Methods:**

A retrospective study was conducted to evaluate DSP patients who visited the emergency department of a tertiary teaching hospital between 2000 and 2011. The data included socio-demographic information, clinical variables, risk factors (e.g., the method used, whether consciousness was impaired, toxicity, reversibility and whether treatment was required) and rescue factors (e.g., location, who initiated the rescue, the probability of discovery, the accessibility of rescue, and delay until discovery). The high-risk group consisted of patients with 11–15 risk points, whereas patients in the low-rescue group had 5–11 risk points. We examined the characteristics of high-lethality suicide attempts (high-risk/low-rescue group).

**Results:**

A total of 1114 patients were enrolled in this study. Pearson’s correlation analysis showed that the total risk score for patients with DSP was negatively associated with the total rescue score (r = −0.201, p < 0.001). Of the total number of DSP patients, 42 were included in the high-risk/low-rescue group. The multivariate logistic regression analyses showed significant associations between high-lethality suicide attempts and male gender (OR 2.70, 95 % CI 1.41–5.18, p = 0.003), older age (OR 1.02, 95 % CI 1.01–1.04, p = 0.015), and unemployment (OR 2.98, 95 % CI 1.41–6.33, p = 0.004).

**Conclusion:**

This retrospective study demonstrates that male gender, advanced age, and unemployed status were associated with high-lethality suicide attempts in DSP patients.

## Background

Suicide is one of the most serious and urgent public health issues in South Korea [1]. South Korea has the highest suicide rate among member countries of the Organization for Economic Cooperation and Development, and suicide was the fourth leading cause of death following cancer, stroke, and cardiovascular disease in 2011 [1].

Attempted suicide is known to increase the risk of subsequent death through suicide [2]. Suicide attempts have been reported to occur 10–20 times more frequently than completed suicides [3]. Different studies have shown that the lifetime prevalence in different countries varies widely, ranging from 1.3 to 6.2 % [4, 5]. Among various suicide methods, deliberate self-poisoning (DSP) is typically classified as a low-lethality method [6, 7], but the majority of self-harm episodes involve poisoning [8]. The risk of subsequent suicide after the first presentation of DSP is more than 40-fold higher in DSP patients compared to population-based controls [9]. Furthermore, one study assessing suicide methods showed that DSP is the most commonly used suicide method in different countries [10].

Suicidal intent refers to the patient’s subjective expectation and desire to die as a result of a self-inflicted injury [11]. However, this expectation sometimes may not correspond to the lethality of an attempt. Some people harm themselves deliberately without suicidal intent. Therefore, it is difficult to predict the risk of a completed suicide based on suicidal intent alone. Suicide risk assessment is a complex process involving the consideration of many factors and contexts, and two separate dimensions must be assessed to identify suicide attempts. These dimensions include the degree of intent to die by suicide and the severity of the self-injury at the time of the attempt. One validated clinical scale, the risk/rescue rating scale (RRRS), was developed to measure the severity of self-injury at the time of the suicide attempt [12]. The RRRS is expressed as a ratio of factors influencing risk and rescue, and the ratio of risk to rescue may be a balance of calculated factors related to the degree of irreversible damage. Of the possible combinations of risk and rescue, those patients in the high-risk/low-rescue group showed suicide attempts with the highest lethality. Kim et al. reported that elderly suicide attempters had high-risk scores and low-rescue scores [13]. However, there has been no study conducted on the variable demographic characteristics of individuals with high-risk and low-rescue scores.

In homogeneous subgroups of DSP, we examined (1) the characteristics of high-risk suicide attempts; (2) the characteristics of low-rescue suicide attempts; and (3) the characteristics of high-risk/low-rescue suicide attempts. In line with Kim et al., we expected that the elderly would be associated with high-risk and low-rescue attempts. Our results provide other factors associated with high-lethality attempts.

## Methods

We retrospectively reviewed the medical records of patients who visited the emergency department (ED) of a tertiary teaching hospital in Seoul, Korea, after a drug overdose between January 1, 2000 and December 31, 2011. We used a researcher-created record form that was based on a toxicology manual and has been in use since 1997. Data from patients who overdosed on drugs but denied suicidal intent were excluded. Additionally, patients with incomplete data were excluded. Two emergency physicians independently reviewed the medical services records and medical records, including the psychiatric records. Any discrepancies were arbitrated by a third investigator. This study was approved by the Institutional Review Board of the Catholic University of Korea, Seoul Saint Mary’s Hospital.

Demographic information and clinical variables such as gender, age, occupation, alcohol co-ingestion with DSP, living with a family member, numbers of previous suicide attempts, and underlying psychotic disorders were evaluated.

The RRRS was used to assess the suicide attempt’s lethality. The psychometric properties of this scale have been previously validated [12]. This scale consists of ten items: five items describe risk factors (e.g., the method used, impairment of consciousness, toxicity, reversibility and treatment required), and five describe rescue factors (e.g., location, person initiating rescue, probability of discovery, accessibility of rescue, and delay until discovery) [12]. Each of the ten items was scored as 1, 2 or 3. These variables and their definitions are listed in Table 1. According to definition of the ‘Agents used’ factor, all DSP patients were given scores of 1 (Table 1).

**Table 1:** Risk-rescue rating

Risk factors	Rescue factors
1. Agent used	1. Location
1. Ingestion, cutting, stabbing	3. Familiar
2. Drowning, asphyxiation, strangulation	2. Non-familiar, non-remote
3. Jumping, shooting	1. Remote
2. Impaired consciousness	2. Person initiating rescue^a^
1. None in evidence	3. Key person
2. Confusion, semicoma	2. Professional
3. Coma, deep coma	1. Passerby
3. Lesions/toxicity	3. Probability of discovery by any rescuer
1. Mild	3. High, almost certain
2. Moderate	2. Uncertain discovery
3. Severe	1. Accidental discovery
4. Reversibility	4. Accessibility to rescue
1. Good, complete recovery expected	3. Asks for help
2. Fair, recovery expected with time	2. Drops clues
3. Poor, residuals expected, if recovery	1. Does not ask for help
5. Treatment required	5. Delay until discovery
1. First aid, E.W. care	3. Immediate 1 h
2. House admission, routine treatment	2. Less than 4 h
3. Intensive care, special treatment	1. Greater than 4 h
Total risk points	Total rescue points

Risk score	Rescue score^b^
5. High risk (13–15 risk points)	1. Least rescuable (5–7 rescue points)
4. High moderate (11–12 risk points)	2. Low moderate (8–9 rescue points)
3. Moderate (9–10 risk points)	3. Moderate (10–11 rescue points)
2. Low moderate (7–8 risk points)	4. High moderate (12–13 rescue points)
1. Low risk (5–6 risk points)	5. Most rescuable (14–15 rescue points)

Each of the ten items were scored as 1, 2 or 3 and the total risk (or rescue) score was calculated by summing the score for each item

^a^Self-rescue automatically yields a rescue score of 5

^b^If there was undue delay in obtaining treatment after discovery, the final rescue score was reduced by one point

The total risk (or rescue) points are calculated by summing the score of each item to achieve a total score of 5–15 points. The total risk (or rescue) points are then converted to an overall risk (or rescue) score ranging from 1 to 5. Each of the five scores match the levels of risk (or rescue) according to the following scale: high risk (risk score 5, 13–15 risk points), high moderate (risk score 4, 11–12 risk points), moderate (risk score 3, 9–10 risk points), low moderate (risk score 2, 7–8 risk points), low risk (risk score 1, 5–6 risk points), least rescuable (rescue score 1, 5–7 rescue points), low moderate (rescue score 2, 8–9 rescue points), moderate (rescue score 3, 10–11 rescue points), high moderate (rescue score 4, 12–13 rescue points), and most rescuable (rescue score 5, 14–15 rescue points) [12]. Higher risk scores indicate more lethal suicide attempts, and higher rescue scores mean less serious and more easily rescued suicide attempts. In the present study, instead of a ratio of risk score to rescue score, we dichotomized patients into the high-risk group (risk score 4, 5) and the low-risk group (risk score 1–3) and into the low-rescue group (rescue score 1–3) and the high-rescue group (rescue score 4, 5). We analyzed the characteristics of the high-risk and low-rescue groups and evaluated the factors associated with these groups. To examine the high lethality of suicides in DSP patients, we also evaluated the factors associated with the DSP patients included in high-risk/low-rescue group.

The distributions of patient baseline characteristics are presented as the means ± standard deviations or as frequencies (i.e., percentages). Categorical variables were compared between groups using the Chi squared test, and continuous variables were compared between groups using the Student’s *t* test. All variables with a significance level p < 0.05 in a univariate analysis were included in a multivariable logistic regression model, and odds ratios (OR) and 95 % confidence intervals (CI) were estimated. In all patients, linear associations between risk scores and rescue scores were assessed using Pearson’s correlation coefficients. All statistical analyses were performed using SPSS version 16.0 (SPSS, Chicago, IL, USA), and p-values less than 0.05 were considered significant.

## Results

**Fig. 1:**
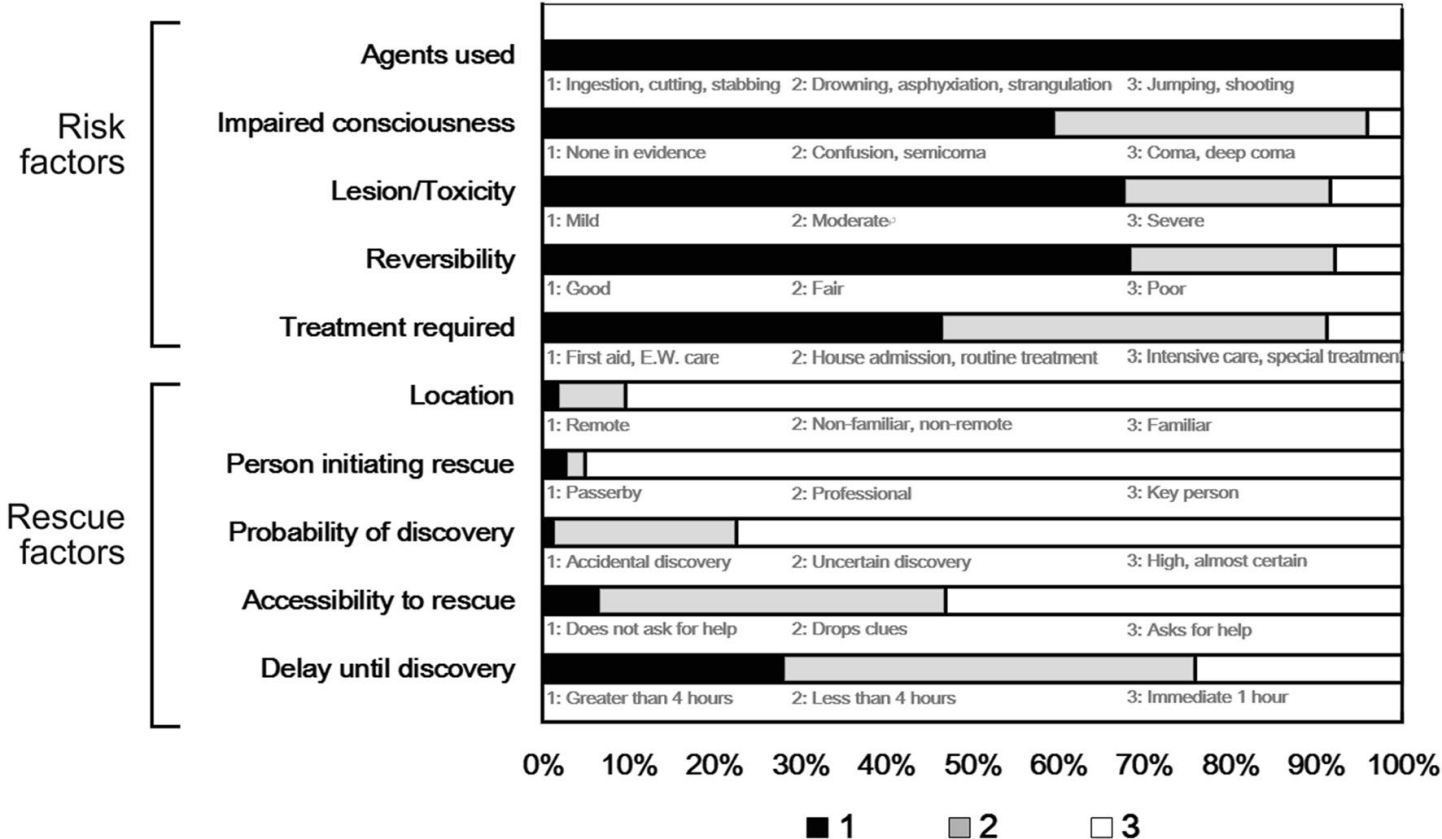
Frequencies of scores for each risk and rescue factor among all patients

A total of 1273 patients were screened from the poisoning database. Of these, 22 patients were excluded due to denying suicidal intent, and 137 patients were excluded from the study due to incomplete data. As a result, a total of 1114 patients were enrolled in this study. Of these patients, 253 (22.8 %) were male and 861 (77.2 %) were female. The mean age of the 1114 patients was 37.17 ± 16.76 years and ranged from 13 to 94 years. The characteristics of the enrolled patients are shown in Table 2. The frequencies of each risk and rescue factors of the patients are shown in Fig. 1. In our cohort, lower risk scores and higher rescue scores were more common than higher risk scores and lower rescue scores. Pearson’s correlation analysis showed that the total risk score of DSP patients was negatively associated with the total rescue score, but the degree of correlation between the two scores was moderate (r = −0.201, p < 0.001).

**Table 2:** Socio-demographic characteristics of study participants

Variables	Value
Male, n (%)	253 (22.7)
Age, years (mean ± SD)	37.17 ± 16.76
Previous attempts, n (%)	248 (22.3)
Unemployed, n (%)	435 (39.0)
Not living with family, n (%)	235 (21.1)
Alcohol co-ingestion, n (%)	352 (31.6)
Psychiatric history, n (%)	294 (26.4)

**Fig. 2:**
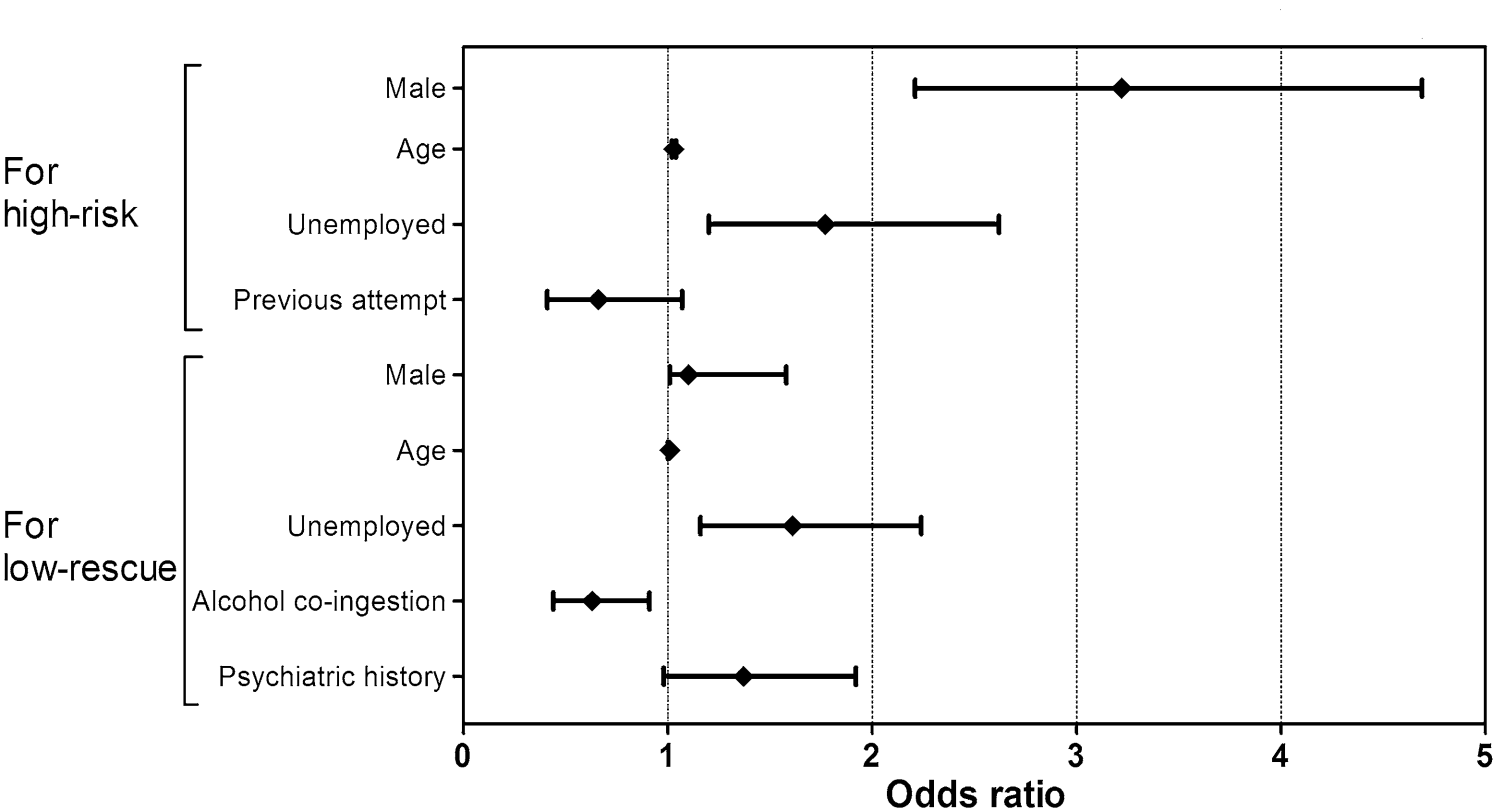
Odds ratio for each factor associated with the high-risk or low-rescue groups in cases of deliberate self-poisoning. Each plot represents the odds ratio and the 95 % confidence interval

In the risk analysis, 156 patients were included in the high-risk group and 958 were included in the low-risk group (Table 3). The rate of death prior to discharge was significantly higher in the high-risk group than in the low-risk group (p < 0.001); only one patient in the low-risk group died prior to discharge. The average ages of the patients in the low-risk and high-risk groups were 35.48 ± 15.70 and 47.57 ± 19.20 years, respectively (p < 0.001). Male gender, previous suicide attempts, and unemployment were also significantly associated with high-risk suicide attempts (p < 0.001, p = 0.043, p < 0.001, respectively). Differences in living with family members, alcohol co-ingesting with DSP, and psychiatric history were not statistically significant between the two groups. In the logistic regression analyses, the high-risk group was significantly associated with male gender (OR 3.22, 95 % CI 2.21–4.69, p < 0.001), older age (OR 1.03, 95 % CI 1.02–1.04, p < 0.001), and unemployment (OR 1.77, 95 % CI 1.20–2.62, p = 0.004) (Fig. 2).

**Table 3:** Comparison of patient socio-demographic characteristics and risk-rescue scores in cases of deliberate self-poisoning

	Risk factors			Rescue factors		
	Low-risk (n = 958)	High-risk (n = 156)	p	Low-rescue (n = 200)	High-rescue (n = 914)	p
Male, n (%)	176 (18.4)	77 (49.4)	<0.001	62 (31.0)	191 (20.9)	0.002
Age, years (mean ± SD)	35.48 ± 15.70	47.57 ± 19.20	<0.001	39.67 ± 19.70	36.63 ± 16.00	<0.001
Previous attempts, n (%)	223 (23.3)	25 (16.0)	0.043	49 (24.5)	199 (21.8)	0.401
Unemployed, n (%)	339 (35.4)	96 (61.5)	<0.001	102 (51.0)	333 (36.4)	<0.001
Not living with family, n (%)	206 (21.5)	29 (18.6)	0.408	43 (21.5)	192 (21.0)	0.877
Alcohol co-ingestion, n (%)	294 (30.7)	58 (37.2)	0.106	50 (25.0)	302 (33.0)	0.027
Psychiatric history, n (%)	262 (27.3)	32 (20.5)	0.072	64 (32.0)	230 (25.2)	0.047
Death prior to discharge, n (%)	1 (0.1)	28 (17.9)	<0.001	10 (5.0)	19 (2.1)	0.019

In the rescue analysis, 200 patients were included in the low-rescue group and 914 patients were included in the high-rescue group (Table 3). Although death prior to discharge was significantly higher in the low-rescue group than in the high-rescue group (p = 0.019), 19 patients (2.1 %) in the high-rescue group died before discharge. Gender, age, unemployment, and alcohol co-ingestion were significantly different between the two groups (p = 0.002, p < 0.001, p < 0.001, p = 0.027, respectively). The between-group differences in previous suicide attempts and living with family were not statistically significant. In the logistic regression analyses, the low-rescue group was significantly associated with male gender (OR 1.58, 95 % CI 1.10–2.26, p = 0.012) and unemployment (OR 1.61, 95 % CI 1.16–2.24, p = 0.004) and negatively associated with alcohol co-ingestion (OR 0.63, 95 % CI 0.44–0.91, p = 0.012) (Fig. 2).

Finally, 42 of the total number of DSP patients showed high-lethality suicide attempts (Table 4). The multivariate logistic regression analyses showed a significant association between high-lethality suicide attempts and male gender (OR 2.70, 95 % CI 1.41–5.18, p = 0.003), older age (OR 1.02, 95 % CI 1.01–1.04, p = 0.015), and unemployment (OR 2.98, 95 % CI 1.41–6.33, p = 0.004) (Table 5).

**Table 4:** Comparison of socio-demographic characteristics and high-lethality suicide attempts

	High-lethality suicide attempts (n = 42)^a^	Non high-lethality suicide attempts (n = 1072)	p
Male, n (%)	22 (52.4)	231 (21.5)	<0.001
Age, years (mean ± SD)	48.57 ± 20.93	36.73 ± 16.43	0.001
Previous attempts, n (%)	7 (16.7)	241 (22.5)	0.374
Unemployed, n (%)	31 (73.8)	404 (37.7)	<0.001
Not living with family, n (%)	11 (26.2)	224 (20.9)	0.409
Alcohol co-ingestion, n (%)	15 (35.7)	337 (31.4)	0.559
Psychiatric history, n (%)	11 (26.2)	283 (26.4)	0.976
Death prior to discharge, n (%)	9 (21.4)	20 (1.9)	<0.001

^a^High-lethality suicide attempts defined as attempts with both high-risk and low-rescue suicide attempts

**Table 5:** Multivariate logistic regression analysis for factors associated with high-lethality suicide attempts in cases of deliberate self-poisoning

	Odds ratio	95 % confidence interval	p
Male	2.70	1.41–5.18	0.003
Age	1.02	1.01–1.04	0.015
Unemployed	2.98	1.41–6.33	0.004

## Discussion

This study showed that in DSP patients, male gender, older age, and unemployment status are associated with high-lethality suicide attempts. Notably, alcohol co-ingestion was significantly associated with high-rescue attempts.

The finding that some factors were associated with high-lethality attempts was in line with previous research and has been consistently reported. In general, men who attempt suicide are known to use more lethal methods, such as firearms or hanging, which often result in severe life-threatening consequences [14, 15]. Among DSP patients, female patients are more common but male patients show more severe poisoning [16–19]. In this study, male gender was significantly associated with both the high-risk and low-rescue groups. A number of factors may have contributed to these gender differences [20]. First, men who are depressed are more likely to have comorbid alcohol and substance abuse problems compared with women, which places men at higher risk. Second, unlike men, who are less likely to seek and accept help or treatment, women are less impulsive, more socially embedded, and more willing to seek help [20, 21].

It is well known that unemployment is a risk factor for suicide, particularly in males [22]. In a study based on a sample of the population of England and Wales, unemployment was shown to be related to a doubling of the suicide rate [23]. The results of this study showed that unemployment was significantly associated with high-lethality DSP.

Age-related psychosocial stressors and family or developmental issues have a direct influence on suicide risk. In our study, the patients in the high-risk and low-rescue groups were older than the patients in the low-risk and high-rescue groups, and older age was also shown to be an independent predictor of a high-lethality suicide attempts. This finding is in line with the findings of a previous study, which showed that elderly suicide attempters had higher risk scores and lower rescue scores [13].

Especially in the rescue analysis, the lower rescue group was negatively associated with alcohol co-ingestion with DSP. This finding is generally inconsistent with those findings published in the literature. It is well known that alcoholism is associated with an increased risk of suicide, and suicide mortality rates for alcoholics are approximately six times those of the general population [2, 24]. Suicide attempts involving alcohol are more likely to be impulsive [25], and people who consume alcohol before attempting suicide are also more likely to use a firearm as a suicide method [26–28]. When we restricted inclusion to DSP patients in this study, there was no significant difference between the high-risk and low-risk groups with regard to alcohol co-ingestion. Interestingly, acute alcohol use with attempted suicide was an independent predictor of high-rescue suicide attempts among DSP patients. Under the influence of alcohol, people are more likely to attempt suicide around other people and to talk about their intent to attempt suicide; these factors are associated with high rescue scores and probably reflect the impulsive nature of suicide attempts related to alcohol use.

The results of this study should be interpreted in the context of several limitations. A major limitation of this study was the retrospective analysis. Because researchers retrospectively reviewed the medical records (including the psychiatric records) and 11 % of the DSP patients refused or could not undergo a psychiatric interview, there was an inevitable risk of bias. Second, this study did not examine the patients’ suicide attempt motives, social class, marital status, substance abuse, or other medical illnesses other than basic socio-demographic characteristics. Finally, this study used a single-center design in the country of South Korea. Nevertheless, this study used data collected over a long period of time (12 years) and included many patients. A large, prospective study including other variables may support our conclusion.

## Conclusions

This retrospective study demonstrated an association between high-lethality suicide attempts and DSP patients who are male, elderly, and have no occupation. However, alcohol co-ingestion before a suicide attempt was significantly associated with high-rescue attempts.
